# Abnormal functional activity in the cerebellar crus can distinguish patients with migraine with comorbid insomnia

**DOI:** 10.3389/fnins.2026.1745862

**Published:** 2026-01-22

**Authors:** Yingsheng Zhang, Changlin Wang, Wei Gui, Xiaojun Feng, Yu Wang

**Affiliations:** 1 Department of Neurology, The First Affiliated Hospital, Anhui Medical University, Hefei, China; 2 Department of Neurology, The Fifth Affiliated Hospital, Anhui Medical University, Fuyang, China; 3 Department of Rehabilitation Medicine, The Fifth Affiliated Hospital, Anhui Medical University, Fuyang, China; 4 Department of Neurology, The Third Affiliated Hospital, Anhui Medical University, Hefei, China; 5 Department of Neurology, The First Affiliated Hospital of USTC, Life Sciences and Medicine Division, University of Science and Technology of China, Hefei, China; 6 Department of Rehabilitation Medicine, The Second Affiliated Hospital, Anhui Medical University, Hefei, China

**Keywords:** amplitude of low-frequency fluctuations, cerebellum crus, functional connectivity, insomnia, migraine

## Abstract

**Background:**

Migraine is a prevalent neurological disorder that is frequently observed in clinical practice and is commonly comorbid with insomnia. Insomnia can exacerbate and precipitate migraine attacks, with both conditions exerting a reciprocal influence on one another. The cerebellar crus is significantly associated with the pathophysiology of migraine and insomnia. The relationship between cerebellar crus functional alterations and migraine-associated insomnia remains unclear. This study utilizes resting-state functional magnetic resonance imaging (rs-fMRI) to examine functional alterations in the cerebellar crus of patients with migraine and concurrent insomnia.

**Methods:**

Participants underwent resting-state functional magnetic resonance imaging. Subsequently, the disparity in amplitude of low-frequency fluctuations (ALFF) values among groups was analyzed, followed by functional connectivity (FC) investigations employing the cerebellum crus as seed regions.

**Results:**

Migraine patients frequently experience neuropsychological disorders and insomnia, which are interconnected. Both migraine with insomnia (MwI) and migraine without insomnia (MwoI) groups demonstrated elevated amplitude of low-frequency fluctuations (ALFF) in the left Crus I and II compared to the healthy controls (HC) group, with the MwI group exhibiting more pronounced alterations. Additionally, both patient groups showed decreased FC between the left Crus I and the right middle temporal gyrus (MTG) and inferior temporal gyrus (ITG) relative to the HC group. The MwoI group showed significantly lower FC compared to both the HC and MwI groups. A significant negative correlation was observed between ALFF in the left Crus I/II and Pittsburgh Sleep Quality Index (PSQI) scores in the MwoI group. Conversely, in the combined migraine cohort, FC between the left Crus I and the right MTG/ITG showed a positive correlation with PSQI scores.

**Conclusion:**

This study identified a correlation between aberrant functional activity in the left Crus I/II and migraine comorbidity with insomnia. These findings provide fresh perspectives on the neural mechanisms underlying the migraine-insomnia relationship, thereby facilitating the identification of potential neuroimaging biomarkers and the exploration of targeted interventions for this patient subgroup.

## Introduction

Migraine, a persistent neurological condition, affects about one billion individuals globally and ranks as the second greatest cause of disability across all age demographics, with a greater prevalence among women. Migraines often co-occur with other conditions, such as insomnia, which can both trigger and exacerbate migraine attacks. Quality sleep can alleviate migraine episodes. Nonetheless, the brain mechanisms connecting migraines and insomnia remain largely unexplored (Nahman-Averbuch et al., 2022). Resting-state functional magnetic resonance imaging (rs-fMRI) has been widely adopted to investigate the neural correlates of both migraine and insomnia. Accumulating evidence points to an important role for the cerebellum in migraine pathophysiology. Specifically, the cerebellum contributes to pain processing and analgesic pathways (Wang et al., 2022; Dobos et al., 2023), participates in the pain matrix network (Lee et al., 2019; Chen et al., 2022; Vasiliou et al., 2024), and has been proposed as a potential imaging biomarker for the risk of migraine chronification (Messina et al., 2021). The bilateral posterior cerebellar lobes, in particular, are implicated in these mechanisms (Fu et al., 2022). Within these regions, the cerebellar crus has been identified as a key structure involved in the inhibitory modulation of nociception (Mehnert and May, 2017).

Parallel research has established a link between the cerebellum and insomnia. Crus I/II, located in the posterior lobe, are thought to modulate arousal and sensory prediction via functional coupling with the thalamus (Guo et al., 2023; Li et al., 2021; Ni et al., 2024). Given these distinct lines of evidence, the posterior cerebellum—and the crus in particular—has been hypothesized as a potential shared neural substrate in the comorbidity of migraine and insomnia. Nevertheless, direct neuroimaging evidence supporting this hypothesis remains absent.

This study employed amplitude of low-frequency fluctuations (ALFF) to detect local variations in brain functional activity, followed by seed-based functional connectivity (FC) analyses using bilateral cerebellar Crus I and II as seeds to examine differences in intrinsic FC networks among migraine subtypes. We aimed to characterize alterations in cerebellar functional properties in migraineurs with or without insomnia to examine their correlation with sleep disturbances.

## Materials and methods

### Participants

This study ultimately enrolled eighty-nine right-handed participants of Han Chinese ethnicity. All participants were recruited from and completed data collection procedures at The Fifth Affiliated Hospital, Anhui Medical University. The final study cohort comprised thirty-one healthy controls (HCs) and fifty-eight patients clinically diagnosed with migraine without aura (MwoA). According to standardized insomnia assessment criteria, the MwoA patients were further categorized into two clinical subgroups: twenty-four with comorbid insomnia (MwI) and thirty-four without insomnia (MwoI). All enrolled participants successfully completed the standardized neuropsychological assessment battery and rs-fMRI scans. The diagnostic criteria for MwoA are derived from the International Classification of Headache Disorders, Third Edition (ICHD-3). Insomnia was identified according to the International Classification of Sleep Disorders, third edition (ICSD-3), with a Pittsburgh Sleep Quality Index (PSQI) score greater than seven serving as a supportive criterion. Subsequently, all enrolled subjects underwent a series of neuropsychological assessments and resting-state functional magnetic resonance imaging (rs-fMRI) scans. Prior to the imaging session, all participants completed a battery of neuropsychological assessments, including the Patient Health Questionnaire-9 (PHQ-9), Pittsburgh Sleep Quality Index (PSQI), and Generalized Anxiety Disorder-7 (GAD-7) scales. Additionally, migraine patients provided detailed clinical characteristics pertaining to the preceding 3 months: headache frequency (days per month), headache intensity assessed by the Visual Analog Scale (VAS), attack duration (hours), and the level of headache-related disability measured by the Migraine Disability Assessment (MIDAS) questionnaire. The exclusion criteria were (1) a history of smoking, alcohol abuse, or excessive coffee consumption; (2) a diagnosis of any neurological or psychiatric disorder other than migraine; (3) use of sedatives, anxiolytics, antidepressants, or prophylactic migraine medications within the past week or 3 months, respectively; and (4) any contraindications to magnetic resonance imaging (MRI). Furthermore, the HCs were required to exhibit satisfactory sleep quality and stable mood, in addition to meeting all the exclusion criteria applied to the patient groups.

### Imaging data acquisition

The MRI scans were conducted with a Siemens Verio 3.0-Tesla machine with a 64-channel head coil from Siemens Corporation, Erlangen, Germany. T1-weighted volumes of high resolution were acquired via a gradient-echo sequence with a TR/TE value of 2200/2.46 ms, an 8-degree flip angle, a 230 × 230 mm field of view, a 256 × 256 acquisition matrix, and a 0.9 mm slice thickness without an interslice gap. Each volume comprised 208 contiguous slices and was acquired with a single excitation. Resting-state BOLD data were acquired over 8.17 min using echo-planning scans with a TR/TE value of 2000/30 ms, an 80-degree flip angle, a 96 × 96 acquisition matrix, and a 2 mm slice thickness without interslice gaps. A total of 84 interleaved slices were captured using simultaneous multislice imaging with a multiband factor of 3, resulting in 240 volumes per run. During the exam, participants were instructed to close their eyes, remain awake, and utilize foam earplugs to alleviate discomfort caused by noise.

### First data processing

Using the rs-fMRI Data Analysis Toolkit Enhanced Version 1.28 (RESTplus 1.28) and the twelfth version of the Statistical Parametric Mapping software (SPM12), imaging data were processed. Data preprocessing includes discarding the initial 10 volumes, slice timing, correcting motion, normalizing to MNI space using DARTEL, applying linear detrending to eliminate confounding covariates (global signals, white matter, cerebrospinal fluid, and six head motion parameters), filtering in the 0.01–0.08 Hz range, and spatial Gaussian smoothing (FWHM = 6 mm). Subjects exhibiting head movement exceeding 2 millimeters or 2 degrees in any direction were excluded.

### The ALFF and FC analysis

The Fourier transform algorithm is applied to compute the power spectrum’s square root, named the Amplitude of Low Frequency Fluctuation (ALFF), for each voxel within the 0.01–0.08 Hz range, which is conventional in ALFF analysis to reflect meaningful spontaneous brain activity while minimizing physiological noise. Each voxel’s ALFF gets normalized through dividing it by the mean ALFF of the entire brain, producing a normalized ALFF map. For statistical comparison, each subject’s ALFF map is converted to z-scores by centering on the whole-brain mean and scaling with the voxel-level whole-brain standard deviation. After concluding the ALFF analyses, we identified the bilateral cerebellar regions Crus I (MNI coordinates ±46, −58, −30) and Crus II (MNI coordinates ±26, −84, −34) as regions of interest (ROIs) in accordance with previous research (Amin et al., 2017). The average time course of the ROIs was correlated with that of the remaining brain voxels, followed by correlation analysis. The correlation coefficients were standardized with Fisher’s Z transformation.

### Statistical analysis

Statistical analyses were conducted utilizing SPSS (version 27.0), with a significance criterion set at *p* < 0.05. Differences among groups in continuous demographic and neuropsychological factors were assessed throughout the three cohorts utilizing a one-way ANOVA. Chi-square tests were conducted for categorical variables, incorporating age, gender, and education as covariates. In the migraine cohort, partial correlation analysis was utilized to evaluate the relationships between headache features and cognitive indicators. An ANOVA model executed in SPM12 was used for voxel-wise comparisons of ALFF and FC maps, succeeded by paired post-hoc tests (Bonferroni-corrected) to discern unique inter-group differences. These comprehensive brain models were calibrated for gender, age, educational attainment, and ratings of anxiety and depression. The statistical threshold was defined by a voxel-level significance of *p* < 0.001, in conjunction with a cluster-level family-wise error (FWE) correction set at FWE *p* < 0.05.

Furthermore, partial correlation analyses were conducted to examine the relationships between aberrant brain activity/connectivity and clinical traits in the combined migraine cohort and separately within the MwoI and MwI subgroups. All analyses controlled for gender, age, and education, as well as headache frequency, disease duration, attack duration, headache intensity (VAS), and headache-related disability (MIDAS) scores (*p* < 0.05). The mean time series were extracted from significant clusters identified through the above whole-brain analysis using the RESTplus V1.28 toolbox. This software computes the average signal within each FWE-surviving cluster and subsequently applies a Fisher’s Z-transformation.

## Results

### Characteristics of demographics and clinical aspects

**Figure 1:**
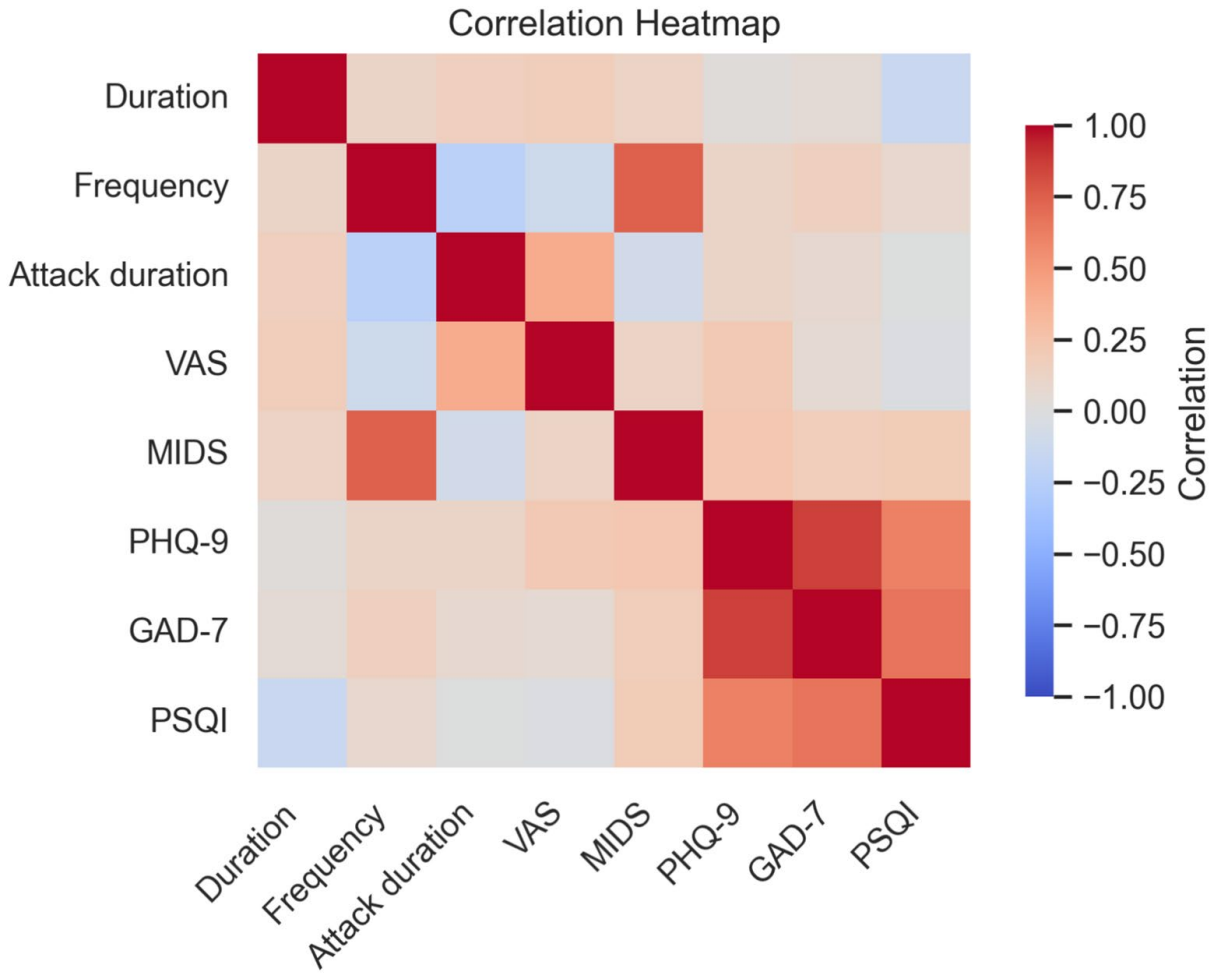
The correlation matrix of clinical features in the combined migraine groups. Abbreviations match those listed in Table 1.

Eighty-nine participants meeting inclusion criteria were analyzed (Table 1). When comparing clinical and demographic factors among the three groups, no significant differences in gender, age, or educational achievement were observed; significant discrepancies were noted in PHQ-9, GAD-7, and PSQI scores (*p* < 0.001), with *post hoc* analysis revealing that the MwI group’s scores exceeded those of the other groups. Correlation between migraine characteristics and PHQ-9, GAD-7, and PSQI scores within pooled migraine (MwI and MwoI) patients: These significant positive correlations were observed: MIDAS scores with headache frequency (r = 0.74, *p* < 0.001), attack duration with VAS scores (r = 0.41, *p* = 0.002), GAD-7 with PHQ-9 scores (r = 0.87, *p* < 0.001), and PSQI scores with both PHQ-9 (r = 0.62, *p* < 0.001) and GAD-7 scores (r = 0.67, *p* < 0.001), as shown in Figure 1.

**Table 1:** Clinical characteristics and demographics of all participants.

Variable	HC	MwI	MwoI	F/X^2^ value	*p*-value
Education (years)	13.16 ± 2.45	12.47 ± 2.47	11.96 ± 2.55	1.64	0.2
Gender (F/M)	28/3	31/3	24/0	2.39	0.3
Age	32.55 ± 6.56	31.5 ± 6.57	33.04 ± 5.85	0.452	0.64
Duration (years)	/	6.53 ± 6.11	5.83 ± 3.8	0.493	0.21
Frequency (Days/Month)	/	4.06 ± 3.92	4.5 ± 4.68	−0.39	0.43
Attack duration (Hours/Time)	/	15.82 ± 19.69	15.67 ± 15.66	0.03	0.55
VAS	/	5.88 ± 1.25	6 ± 1.77	−0.3	0.22
MIDAS	/	1.91 ± 0.97	2.08 ± 1.06	−0.64	0.81
PHQ-9	1.48 ± 0.89	3.35 ± 2.97	8.58 ± 4.87	35.62	<0.001
GAD-7	1.29 ± 0.64	2.12 ± 2.63	7.71 ± 4.68	36.99	<0.001
PSQI	4 ± 1.29	4.5 ± 1.62	9.92 ± 2.1	101.87	<0.001

MwI, migraine with insomnia, MwoI, migraine without insomnia, HC, healthy control. VAS, Visual Analog Scale; MIDAS, Migraine Disability Assessment Score; PSQI, Pittsburgh Sleep Quality Index; PHQ-9, Patient Health Questionnaire-9; GAD-7, Generalized Anxiety Disorder-7.

### ALFF

**Figure 2:**
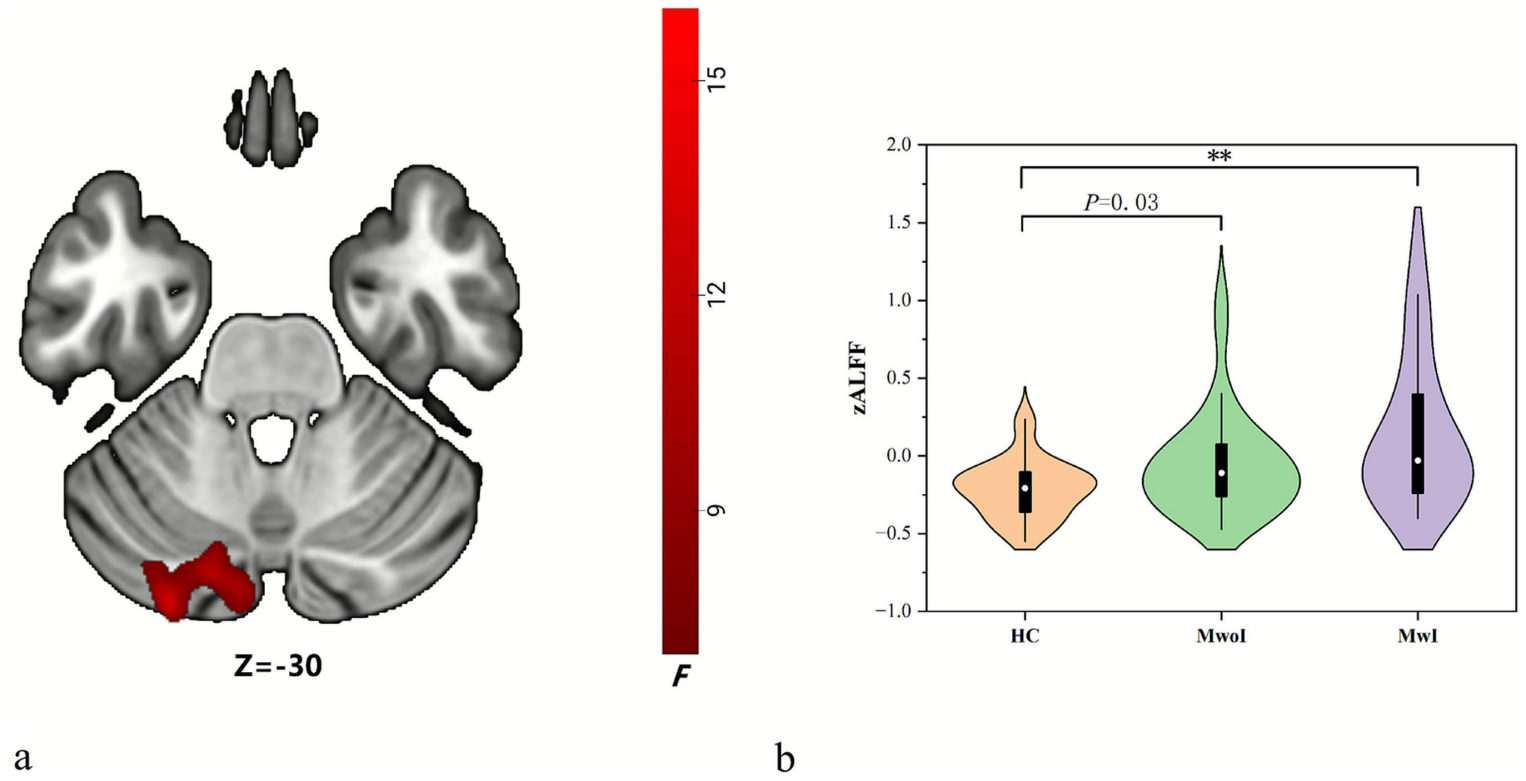
Distinct brain regions in ALFF among all groups. The brain map **(a)** reveals differences in ALFF (red) within the left crus I and II, and the violin plots **(b)** display their ALFF values across the three groups, with ** indicating *p* < 0.001 (Bonferroni correction). Abbreviations match those listed in Tables 1, 2.

A one-way ANOVA indicated a significant difference in ALFF values in the left Crus I and II (*F* = 7.17, *p* = 0.001) among groups. Post-hoc tests indicated significantly elevated ALFF levels in both the MwoI group (*p* = 0.03) and the MwI group relative to the HC group (Table 2; Figure 2). The MwI group demonstrated a larger effect size (Bonferroni-corrected *p* < 0.001).

**Table 2:** Differential regions in ALFF and FC Networks.

Measure	Brain region	Cluster size	MNI coordinates			Peak F score
			X	Y	Z	
ALFF	L-CrusI/II	86	−27	−87	−30	14.27
FC	R-MTG/ITG	98	45	−69	0	14.61

ALFF, amplitude of low-frequency fluctuations; FC, functional connectivity; Crus, cerebellum crus; MTG, Middle Temporal Gyrus; ITG, Inferior Temporal Gyrus; R, right; L, left.

### FC analysis from seeds

**Figure 3:**
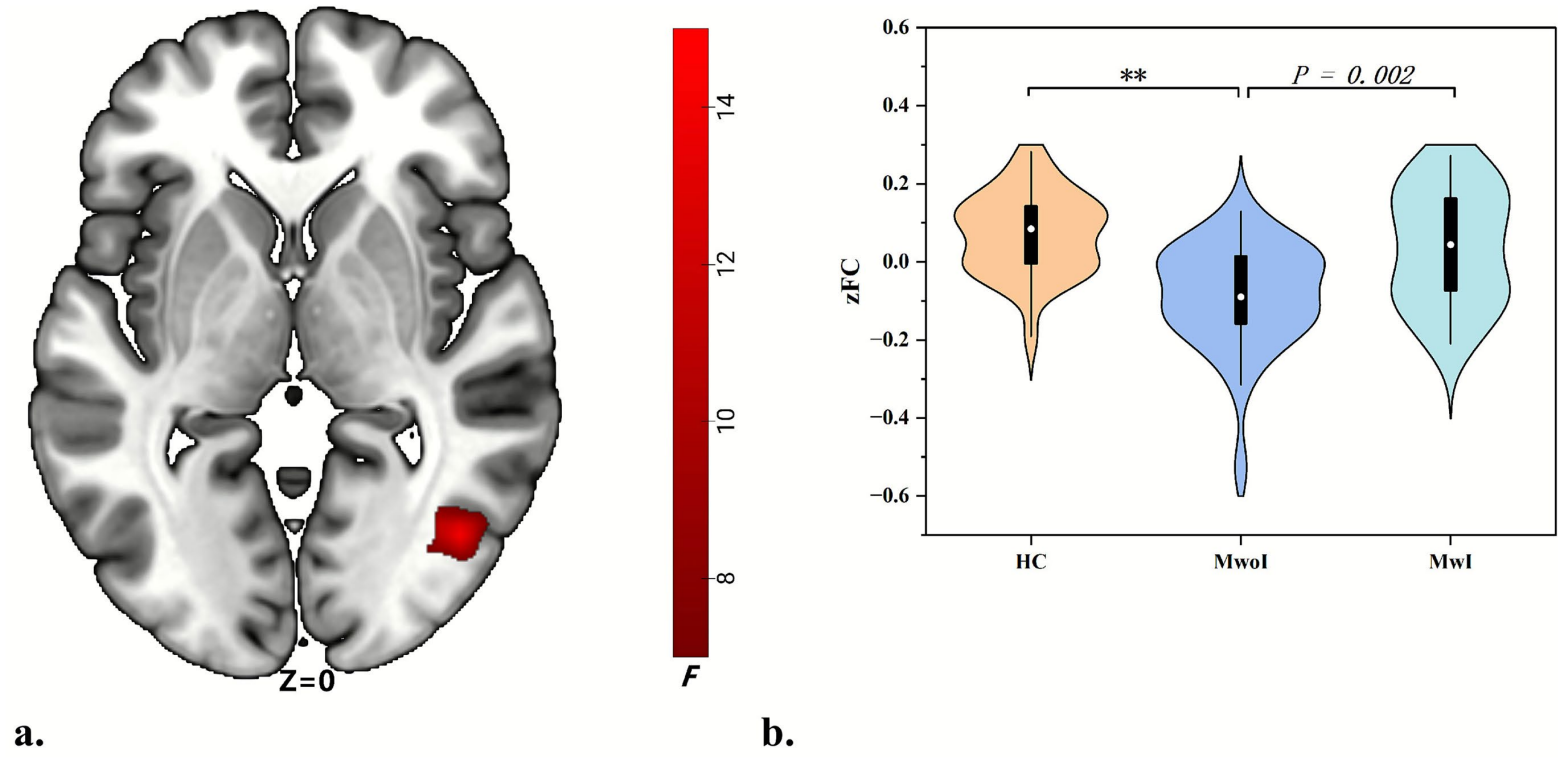
The left crus I FC network exhibits differences across three groups. **(a)** The brain map reveals differences in the right MTG and ITG. **(b)** The violin plots display its FC values across the three groups, with ** indicating *p* < 0.001 (Bonferroni correction). Abbreviations match those listed in Tables 1, 2.

In comparison to the HC group, both the MwoI and MwI groups demonstrated diminished FC intensity; however, only the MwoI group displayed a statistically significant difference (Bonferroni-corrected *p* < 0.001). In comparison to the MwoI group, the MwI group exhibited markedly increased FC intensity (Bonferroni-corrected *p* = 0.002) (Table 2; Figure 3). The FC analyses revealed no statistically significant differences when using the left Crus II, right Crus I, and right Crus II as seed points, respectively.

### Correlation analyses

#### ALFF

In the MwoI group, a negative correlation was identified between the ALFF value of Left Crus I and II and the PSQI (r = −0.41, *p* = 0.04, uncorrected), and in the MwI group, it was identified with PHQ-9 (r = −0.48, *p* = 0.05, uncorrected) and GAD-7 (r = −0.54, *p* = 0.03, uncorrected). In the combined migraine group, the correlation analysis revealed no abnormalities.

#### FC

**Figure 4:**
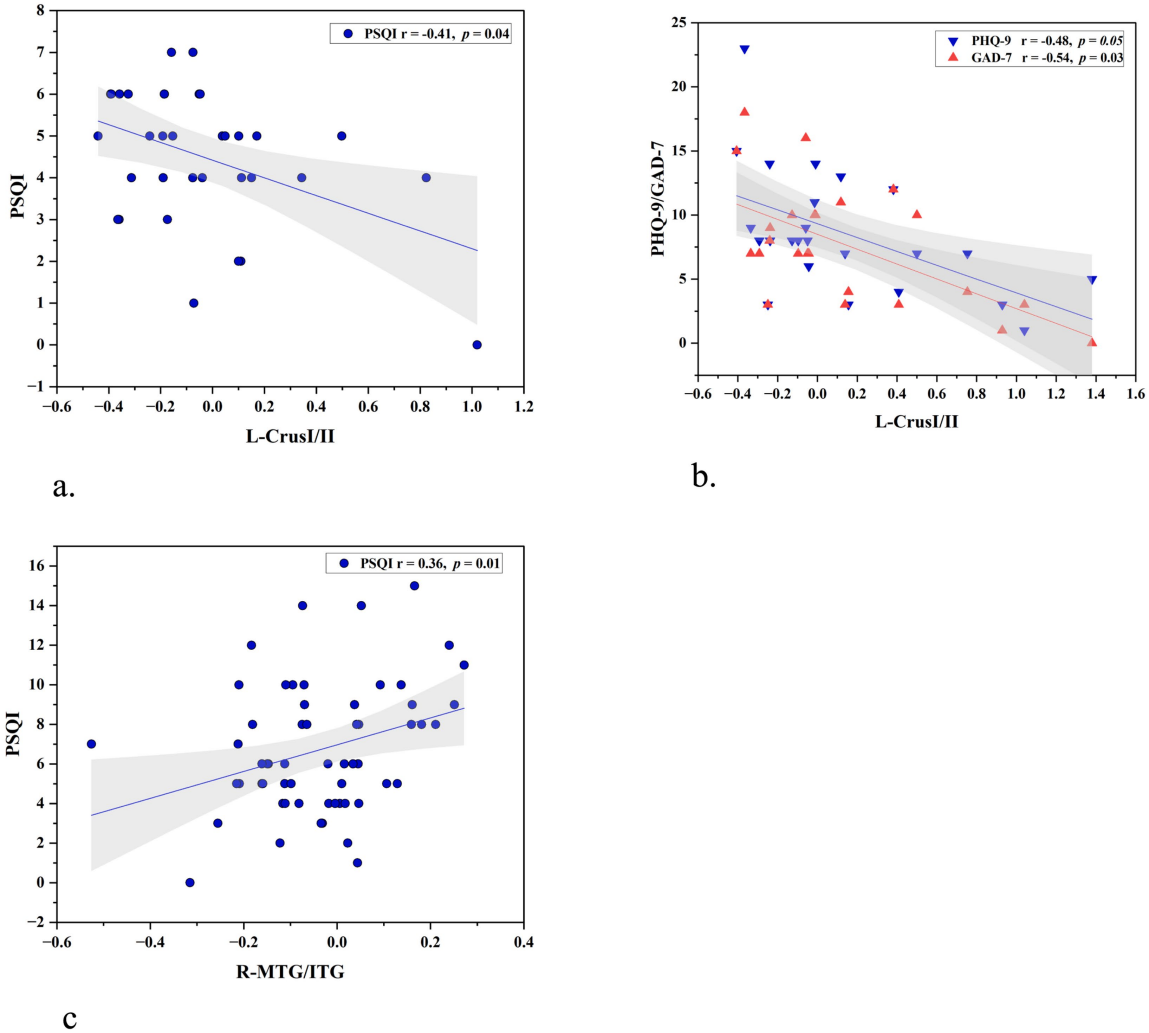
Relationships of cerebellar ALFF and FC with clinical characteristics. **(a)** A significant negative correlation was observed between the ALFF of the left Crus I/II and PSQI scores in the MwoI group. **(b)** In the MwI group, ALFF in the same region was negatively correlated with PHQ-9 and GAD-7 scores. **(c)** Analysis of the combined migraine groups revealed that the FC between the left crus I and the right MTG/ITG was positively correlated with PSQI scores. Abbreviations match those listed in Tables 1, 2.

In the combined migraine group, the connectivity between the left Crus I and the right MTG and ITG was positively related to PSQI scores (r = 0.36, *p* = 0.01, uncorrected). In the MwoI and MwI groups, the correlation analysis revealed no abnormalities. as shown in Figure 4.

## Discussion

This study delineated FC modifications in patients with migraine with MwI and MwoI. Compared to the HC group, both patient cohorts demonstrated heightened ALFF in the left cerebellar Crus I/II, with the MwI group exhibiting a more significant increase. Seed-based FC analysis revealed abnormal connection between the left Crus I and the right temporal lobe, particularly the middle temporal gyrus (MTG) and inferior temporal gyrus (ITG). In traditional brain network classifications, the middle temporal gyrus (MTG) is part of the ventral visual network, whereas the inferior temporal gyrus (ITG) is associated with the default mode network (DMN). While FC in this route was diminished in both patient cohorts relative to the HC group, the decrease was most pronounced in the MwoI group. Exploratory, uncorrected correlation analyses suggested that elevated ALFF in the left Crus I/II might be associated with milder insomnia, while higher FC in the left Crus I showed a positive association with more severe insomnia.

In a cohort of migraine patients, the PSQI scores were positively correlated with both the GAD-7 and the PHQ-9 scores. Additionally, attack duration was positively correlated with the VAS scores, while the MIDAS scores were positively correlated with headache frequency. Migraine patients frequently experience comorbid neuropsychological disorders and insomnia, which can contribute to the exacerbation of their symptoms (Lampl et al., 2016). Therefore, strategies for emotional management and sleep improvement are particularly important for individuals with migraines.

Traditionally linked to motor control, the cerebellum is now recognized as integral to migraine-related pain transmission and analgesic pathways. Migraine sufferers show heightened local functional connectivity density (FCD) in the cerebellum, inversely related to pain intensity. Temporal dynamic irregularities in the cerebellum (mostly in bilateral crus) among migraine sufferers are uncovered by Dynamic fALFF. These irregularities are linked to the clinical severity, indicating that the cerebellum serves as a crucial modulation hub in the migraine network (Chen et al., 2022). The increased connection between the hypothalamus and the cerebellum (primarily in the bilateral crus) can serve as a candidate imaging biomarker for migraine severity and chronicity risk (Messina et al., 2021). Fu’s study has demonstrated abnormal cerebral perfusion in migraine patients, primarily involving the bilateral crus, which correlates with clinical indicators (Fu et al., 2022). The activity in the crus is heightened under pain stimulation and exhibits cognitive and emotional features, which are related to pain encoding (Moulton et al., 2011). The cerebellum predominantly exerts an inhibitory effect on nociception. The activation of the crus is more pronounced in migraine patients compared to control subjects, serving to sustain its inhibitory function (Mehnert and May, 2017). The aforementioned studies all support the notion that the cerebellum, particularly the crus region, possesses a pain regulation mechanism in migraines.

The cerebellum is involved in sleep regulation by modulating monoaminergic neurotransmission and is closely connected to the sleep–wake cycle. Among individuals with chronic insomnia, the FC between the ascending arousal network and cerebellar networks, as well as the internal connections within the cerebellar networks, is enhanced (Gong et al., 2022; Lin et al., 2023). Cerebellar lobules, mostly located in the posterior lobe (especially in Crus, VI, VIIb, and VIII) or the vermis, participate in arousal regulation and sensory prediction through coupling with the thalamus (Guo et al., 2023). Cerebellar gray matter volume, especially in the posterior lobe, changes gradually as insomnia severity increases. Increased gray matter in the right Crus II may represent a compensatory mechanism (Li et al., 2021). The bilateral crus exhibits significant alterations in FC network with the frontoparietal following sleep deprivation (Ni et al., 2024). The bilateral crus serves as a crucial network node for motor interventions aimed at improving sleep (Chen et al., 2024).

Our study found heightened local brain activity in the left crus among migraine patients, with ALFF values negatively correlating with anxiety, depression, and sleep disorder scale scores. This data supports the modulatory role of the cerebellum, specifically the crus, in migraineurs experiencing insomnia. However, this regulatory mechanism is impaired. Abnormal functional activity in the left crus is a key pathological mechanism shared by migraine patients with insomnia, highlighting the cerebellar crus as a crucial focus in brain functional studies of migraine.

The MTG and ITG are higher-order visual processing regions where individuals with migraines display anomalies in visual perception (Niddam et al., 2015). The MTG is acknowledged as a vital brain region for the synthesis of visual and aural inputs. In migraine sufferers, the MTG demonstrates increased neuronal synchronization when exposed to negative audiovisual stimuli in contrast to healthy individuals (Klamer et al., 2025). Individuals with insomnia demonstrate diminished ALFF and ReHo in the right MFG, which coincides with memory deficits and cognitive deterioration in those affected by insomnia (Thomas et al., 2000). The MTG is implicated in the encoding and retrieval of dreams during sleep and plays a crucial role in the processes of emotional episodic memory (Cipolli et al., 2016).

The ITG intersects with the lateral temporal branch of the DMN and is crucial in the neuro-limbic pain modulation network (Chen et al., 2017). In comparison to the HC group, the MWoA group had diminished integrative capacity of the right inferior temporal lobe within whole-brain networks, potentially resulting in deficits in visual processing, memory integration, and multisensory information processing (Gao et al., 2015). ITG dysfunction is associated with the severity of insomnia, as functional abnormalities result in increased physiological arousal in patients with insomnia throughout both diurnal and nocturnal periods (Gong et al., 2020).

Our study observed diminished FC between the left Crus I and the right MTG and ITG in individuals suffering migraines compared to the HC group. Critically, FC values correlated positively with sleep disorder severity even after accounting for potential confounders. The findings indicate that the FC network between the left crus and the VN and DMN is imbalanced and reorganized in individuals with comorbid migraine and insomnia. The altered FC networks result in compromised neuronal regulation mechanisms of the crus towards the VN and DMN. This indicates the same pathophysiological mechanism and biological marker for insomnia linked to migraines. This mechanism underscores the cerebellar crus as a prospective therapeutic target, indicating that neuromodulatory techniques (e.g., transcranial magnetic stimulation) designed to normalize crus-temporal connectivity may provide an innovative treatment strategy for concurrently alleviating both conditions.

### Limitations and expectations

This research has some limitations. The cross-sectional methodology of this study hinders the determination of a causal relationship between migraine, alterations in functional connectivity networks, and local cerebellar dysfunction. Future longitudinal studies will be necessary to validate this. Secondly, the limited sample size led to the absence of some relevant capabilities. Research in the future ought to concentrate on increasing the number of samples. Thirdly, the correlational analyses were exploratory and not corrected for multiple comparisons. Therefore, these uncorrected data should be considered hypothesis-generating rather than confirmatory. The assessment of the sleep scale is subjective, perhaps resulting in grouping bias. Future study should employ objective methods for assessing insomnia, such as polysomnography. Finally, the migraine cohort was not additionally categorized by subtype or onset period. Future analysis must take these things into account.

## Conclusion

Our research has identified abnormalities in the local functional activity within the crus region and aberrant FC networks in migraine patients with comorbid insomnia. This discovery offers novel neuroimaging biomarkers for elucidating the mechanisms underlying migraine with comorbid insomnia. Abnormal localized activity in the left crus, along with its aberrant FC with the right MTG and ITG, is associated with sleep disorders in migraine patients with comorbid insomnia. These findings could be valuable for identifying therapeutic targets for migraine-associated insomnia in future research.

## Data Availability

The datasets presented in this article are not readily available because the data supporting this study’s findings contain sensitive personal information of migraine patients. Due to ethical restrictions and participant confidentiality agreements protected by the Ethics Committee of the Fifth Affiliated Hospital of Anhui Medical University, the raw dataset is not publicly available. Requests to access the datasets should be directed to YZ 13339293378@163.com.
